# The Role of Mustelids in the Transmission of *Sarcocystis* spp. Using Cattle as Intermediate Hosts

**DOI:** 10.3390/ani11030822

**Published:** 2021-03-15

**Authors:** Petras Prakas, Linas Balčiauskas, Evelina Juozaitytė-Ngugu, Dalius Butkauskas

**Affiliations:** Nature Research Centre, Akademijos Str. 2, LT-08412 Vilnius, Lithuania; linas.balciauskas@gamtc.lt (L.B.); evelina.ngugu@gamtc.lt (E.J.-N.); dalius.butkauskas@gamtc.lt (D.B.)

**Keywords:** *Sarcocystis*, cattle, mustelidae, life cycle, *cox1*, molecular identification

## Abstract

**Simple Summary:**

Members of the genus *Sarcocystis* are worldwide distributed protozoan parasites. *Sarcocystis* infections cause great losses in economically important animals. There is a lack of studies on *Sarcocystis* in naturally infected wild predators, especially of the family Mustelidae which represent a presumably important group of definitive hosts of these parasites. The objective of the present study was to examine the small intestine samples of various mustelid species from Lithuania serving as a possible source of *Sarcocystis* spp. using cattle as intermediate hosts. Overall, 84 samples collected from five mustelid species were analyzed. Oocysts/sporocysts of *Sarcocystis* spp. were detected in 75 animals (89.3%). Using molecular methods four *Sarcocystis* spp., *S*. *bovifelis*, *S*. *cruzi*, *S*. *hirsuta* and *S*. *hominis* were identified, with the first two being the most prevalent. These results indicate that mustelids are involved in the transmission of *Sarcocystis* spp. using cattle as intermediate hosts. The determined high prevalence of *Sarcocystis* spp. rates cause concerns about food safety issues. To control the spread of infection, further studies on the way carcasses of cattle or beef waste become accessible to mustelids are needed.

**Abstract:**

There is a lack of research on the role of mustelids in the transmission of various *Sarcocystis* spp. In the present study we tested the hypothesis that widespread mustelids in Lithuania could be involved in the transmission of *Sarcocystis* spp. using cattle as intermediate hosts. In 2016–2020, intestinal samples of 84 mustelids were examined. *Sarcocystis* spp. were identified by species-specific PCR targeting the *cox1* gene and subsequent sequencing. Under a light microscope, oocysts/sporocysts of *Sarcocystis* spp. were observed in 40 samples (47.6%), while using molecular methods, they were detected in 75 animals (89.3%). Four *Sarcocystis* spp. were identified in the intestinal samples of American mink (*Neovison* *vison*), Beech marten (*Martes foina*), European pine marten (*Martes martes*), European badger (*Meles meles*) and European polecat (*Mustela putorius*). The prevalence of predominant *Sarcocystis* spp., *S*. *bovifelis* (89.3%) and *S*. *cruzi* (73.8%) was significantly higher than that of *S*. *hirsuta* (3.6%) and *S*. *hominis* (1.2%). In an individual sample, most frequently two *Sarcocystis* spp. were identified (69.0%), then a single species (15.5%) and three species (4.8%). The present study provides strong evidence that mustelids serve as definitive hosts for *Sarcocystis* spp. using cattle as intermediate hosts.

## 1. Introduction

Representatives of the genus *Sarcocystis* (Apicomplexa: Sarcocystidae) are cyst forming coccidians with an obligatory prey-predator two-host life cycle. Asexual multiplication with the formation of sarcocysts takes place in the extra-intestinal tissues of the intermediate host (IH), while sexual stages (oocysts-sporocysts) develop in the small intestine of the definitive host (DH) [1]. Predators and scavengers serve as DH for *Sarcocystis* spp., whereas prey animals become IH [2].

Members of the family Mustelidae may act as IH or DH for several *Sarcocystis* spp. The agent of equine protozoal myeloencephalitis, *S*. *neurona* was also detected in the muscles of a fisher (*Martes pennanti*), ferret (*Mustela putorius furo*) and American mink (*Neovison vison*) [3]. Additionally, eight species of *Sarcocystis* have been observed in the muscles of various mustelids [4]. Recently described *S*. *lutrae* [5] was identified in the muscles of several Carnivora families, Canidae, Mustelidae and Procyonidae [5,6,7]. The role of mustelids as DH of *Sarcocystis* spp. has not been investigated [8]. 

Mustelidae is the largest and most diverse family in the order of Carnivora in Lithuania, with nine species present [9]. Representatives of mustelids occur in all habitats, including the urban ones [10,11]. The broad habitat niches of the American mink, the Beech marten (*Martes foina*), European badger (*Meles meles*), European pine marten (*Martes martes*) and European polecat (*Mustela putorius*) are reflected in their diverse diets [10,11]. In general, members of the family Mustelidae are opportunistic predators and their diet consists of birds, various mammals, fish, amphibians, invertebrates, fruits, ungulate carcasses, plants and mushrooms [12,13,14,15,16]. In Lithuania, the food chains of mustelids, including cattle carrion, were not investigated in detail, with exception of the European pine marten [17]. Diet of this species in the cold period included 5.3% of carcasses of domestic animals according to the biomass consumed. Thus, far no studies on the role of mustelids in the transmission of *Sarcocystis* in Lithuania have been undertaken. 

Recently, a high prevalence of *Sarcocystis* spp. in cattle from Lithuania has been recorded [18]. By performing trypsinization of the diaphragm muscles and species-specific PCR targeting the *cox1* (mitochondrial gene encoding subunit 1 of cytochrome c oxidase), *S*. *cruzi* was identified in 96.1% of the samples, *S*. *bovifelis* was detected in 71.6% of the samples, *S*. *hirsuta* was confirmed in 30.4% of the samples and *S*. *hominis* was observed in 13.7% of the samples [19]. Canids are DH for *S*. *cruzi*, humans are DH for *S*. *hominis*, whereas *S*. *hirsuta* and *S*. *bovifelis* are transmitted via felids [19]. The Eurasian lynx (*Lynx lynx*) is the only wild member of the felids in Lithuania [9]. However, this species is not abundant and there were approximately 160 lynx individuals in Lithuania in 2018 [20]. Thus, the high prevalence of *S*. *bovifelis* implies that it is not solely felids that contribute to the spread of this species. Therefore, we put forward the hypothesis that mustelids can act as DH of *S*. *bovifelis*. In order to test the assumption, the aim of the present study was to examine the small intestines of various mustelids from Lithuania for the presence of *Sarcocystis* spp. employing cattle as IH. 

## 2. Materials and Methods

### 2.1. Sample Collection

**Figure 1:**
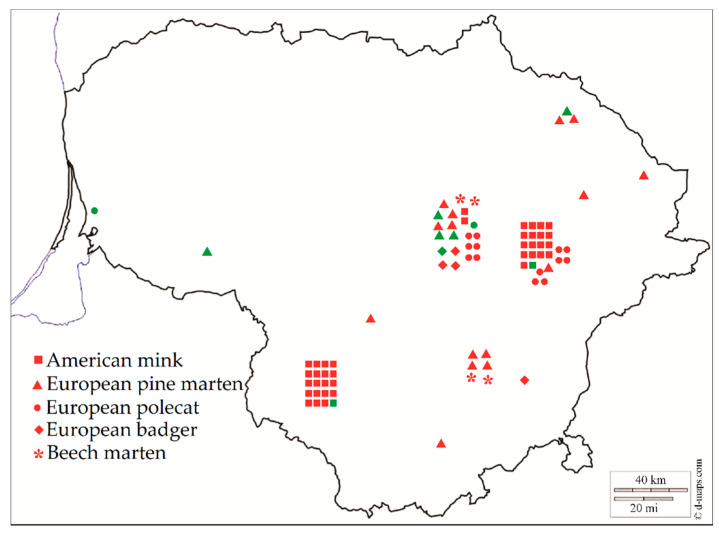
*Sarcocystis* spp. in the species of Mustelidae in Lithuania. Red color means positive individuals, green color represents negative individuals.

Between 2016 and 2020, intestine samples of 84 mustelids (40 American mink, 4 Beech marten, 5 European badger, 20 European pine marten and 15 European polecat) were studied for the presence of *Sarcocystis* spp. The animals were collected from hunters, taxidermists, or biologists who found dead animals on the roadways in different regions of Lithuania (Figure 1). 

### 2.2. Examination of Intestines

Oocysts/sporocysts of *Sarcocystis* spp. were excreted from the entire intestine of each mustelids using a slightly modified Verma et al. [21] technique. At first, faeces of each intestine were squeezed and the entire intestine was cut lengthwise. The intestinal epithelium was lightly scraped with the help of a scalpel blade and suspended in 50 mL of water. The homogenate was centrifuged for 10 min at 1000 rpm, 25 °C in 50 mL centrifuge tubes. The supernatant was discarded and sediments were re-suspended in 50 mL water. Subsequently, the homogenate was centrifuged for 10 min at 1000 rpm, 25 °C and the supernatant was discarded. The examination of the sediments for oocysts/sporocysts under a light microscope was repeated. The 200 μL of re-suspended sediments were taken from each sample and used for DNA extraction. DNA was isolated from all mustelid samples. 

### 2.3. Molecular Analysis

DNA extraction from mucosal suspension was performed using the GeneJET Genomic DNA Purification Kit (Thermo Fisher Scientific Baltics, Vilnius, Lithuania). *Sarcocystis* spp. were identified by nested PCR of partial *cox1* sequences. Primers used in the present study are listed in Table 1. PCRs were conducted in the final volume of 25 μL made of 12.5 μL of DreamTaq PCR Master Mix (Thermo Fisher Scientific, Vilnius, Lithuania), 0.5 μM of each primer, 0.04 μg template DNA and nuclease-free water. The first run of nested PCR began with one cycle at 95 °C for 5 min followed by 35 cycles of 94 °C for 45 s, 58–60 °C, depending on primer pair for 60 s and 72 °C for 80 s and ending with one cycle at 72 °C for 7 min. For the second PCR assay, 1 μL from the first PCR assay was used. Visualization, purification and sequencing of PCR products were carried out using a previously described protocol [22]. The obtained *cox1* sequences were compared with the Nucleotide BLAST program (megablast option) [23]. The *cox1* sequences generated in the present study are available in GenBank with Acc. No. MW595468–MW595608.

### 2.4. Statistical Tests

The prevalence and 95% CI for prevalence were calculated using OpenEpi epidemiological software [25], following the Wilson method for calculating score interval [26]. Differences in the prevalence of the identified *Sarcocystis* spp. were evaluated using the Chi-squared test, calculated in WinPepi, ver. 11.39 and using Upton’s approximation for small and medium sample sizes [27]. Comparing the prevalence of *Sarcocystis* spp., the effect size was expressed according to adjusted Cohen’s w [28].

## 3. Results

### 3.1. Differences in Prevalence of Sarcocystis spp. Using Microscopic and Molecular Methods

Based on microscopic examination, the prevalence of *Sarcocystis* spp. in mucosal scrapings was 47.6% (Table 2). Under a light microscope usually free sporocysts measuring 11.8 × 8.3 µm (7.1–14.5 × 6.5–10.9 μm; *n* = 219) were seen. Sporulated oocysts of *Sarcocystis* 17.7 × 13.1 µm (12.5–23.7 × 10.5–18.3 μm; *n* = 100) were also noticed. With the help of nested PCR and subsequent sequencing *Sarcocystis* spp. were confirmed in 75 animals (89.3%). In general, as compared with morphological examination, the detection rate of *Sarcocystis* spp. was significantly higher (χ^2^ = 33.56, *p* < 0.0001; adjusted Cohen’s w = 0.709, large effect size) when a molecular method was employed. The molecular method yielded significantly more detections in the American mink, European polecat and European badger (Cohen’s w = 1.083, 0.606 and 1.061, respectively, large effect size). Differences between the two methods in the Beech marten and European pine marten were not significant (Table 2). In one American mink and three Beech marten samples, oocysts/sporocysts were detected microscopically, however, these samples were negative for the examined *Sarcocystis* spp. using a molecular analysis. 

Based on molecular analysis, the highest prevalence of *Sarcocystis* spp. was observed in the Beech marten, followed by the American mink and European polecat; however, even the lowest prevalence of *Sarcocystis* spp. detected in the European badger and European pine marten were 75% and higher (Table 2). The prevalence of *Sarcocystis* spp. observed in the Beech marten, American mink and European polecat did not differ statistically (species cluster with the highest prevalence). The prevalence of *Sarcocystis* spp. observed in the American mink was significantly higher (χ^2^ = 5.09, *p* < 0.025; Cohen’s w = 0.435, medium effect size) than that detected in the European pine marten. Other differences were not significant and the effect size was either small or absent.

### 3.2. Molecular Identification of Sarcocystis spp.

The comparison of sequences generated in the present study showed the presence of four *Sarcocystis* spp. (*S*. *bovifelis*, *S*. *cruzi*, *S*. *hirsuta* and *S*. *hominis*) in the analyzed samples of Mustelidae (Table 3).

### 3.3. Distribution of Sarcocystis spp. in the Intestine Samples of Mustelids

**Figure 2:**
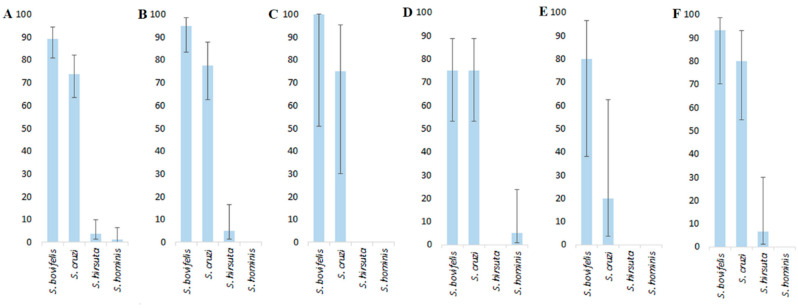
Prevalence of *Sarcocystis* spp. in the examined samples of mustelids. (**A**)—in the pooled sample of all host species, (**B**)—in American mink, (**C**)—in Beech marten, (**D**)—in European pine marten, (**E**)—in European badger, (**F**)—in European polecat. Differences of prevalence in A: *S*. *bovifelis* > *S*. *cruzi* (χ^2^ = 6.65, *p* < 0.01; Cohen’s w = 0.288), >*S*. *hirsuta* (χ^2^ = 123.32, *p* < 0.001; w = 2.376) and >*S*. *hominis* (χ^2^ = 130.79, *p* < 0.001; w = 2.688); *S*. *cruzi* > *S*. *hirsuta* (3.6%, χ^2^ = 86.83, *p* < 0.001; w = 1.472) and >*S*. *hominis* (χ^2^ = 93.94, *p* < 0.001; w = 1.604); in B: *S*. *bovifelis* >*S*. *cruzi* (χ^2^ = 5.10, *p* < 0.025; w = 0.372); in E: *S*. *bovifelis* > *S*. *cruzi* (χ^2^ = 3.24, *p* < 0.075; w = 1.064).

Irrespective of the host species, *S*. *bovifelis* in the examined samples was identified most often (Figure 2A). The prevalence of *S*. *bovifelis* (89.3%) was significantly higher than that of *S*. *cruzi* (73.8%, a small effect size), *S*. *hirsuta* (3.6%, a large effect size) and *S*. *hominis* (1.2%, a large effect size). The prevalence of *S*. *cruzi* was significantly higher than that of *S*. *hirsuta* (3.6%) and *S*. *hominis* (a large effect size both). 

The prevalence of *S*. *bovifelis* was the highest, exceeding that of *S*. *cruzi* in the examined samples of the American mink (a medium effect size, Figure 2B) and European badger (a large effect size, Figure 2E). The prevalence of *S*. *bovifelis* and *S*. *cruzi* did not differ significantly in European polecat (Figure 2F) and Beech marten (Figure 2C); in European pine marten they were equal (Figure 2D). The prevalence of predominant *Sarcocystis* spp., *S*. *bovifelis* and *S*. *cruzi*, was significantly higher than that of *S*. *hirsuta* and *S*. *hominis*, in all host species (Figure 2B–F). Both predominant species were observed in all five examined host species. *Sarcocystis hirsuta* was identified in two American mink individuals and one European polecat individual; whereas *S*. *hominis* was confirmed in one European pine marten individual. 

**Figure 3:**
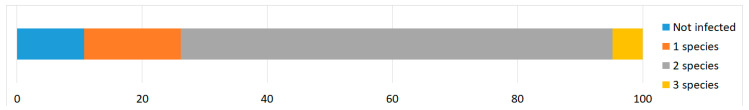
Distribution of the number of *Sarcocystis* spp. identified in the examined samples of mustelids.

Up to three *Sarcocystis* spp. were identified in one host individual (Figure 3). No examined *Sarcocystis* spp. were found in approximately one tenth of the investigated animals (10.7%). The prevalence of single species infections was 15.5%; in all cases when a single species was detected in individual samples, it was *S*. *bovifelis*. Two *Sarcocystis* spp. (69.0%) were most frequently identified in one host individual and in all such cases it was *S*. *cruzi*/*S*. *bovifelis* co-infection. Three *Sarcocystis* spp. were confirmed in four animals (4.8%), one European polecat individual, one European pine marten individual and two American minks. In three of these cases, it was *S*. *bovifelis*/*S*. *cruzi*/*S*. *hirsuta* co-infection, in one case—*S*. *bovifelis*/*S*. *cruzi*/*S*. *hominis* co-infection.

## 4. Discussion

In the present study, high rates (89.3%) of *Sarcocystis* spp. employing cattle as IH were observed in mustelids from Lithuania. Under a light microscope oocysts/sporocysts were detected in 40 out of 84 samples (47.6%). In comparison, the presence of *Sarcocystis* spp. in 75 (89.3%) mucosal scrapings of mustelids were confirmed by molecular methods. Usually, molecular analysis is performed when oocysts/sporocysts of *Sarcocystis* spp. are microscopically detected in intestine mucosal or faecal samples [2,29,30,31]. However, the results of the present study reveal that molecular methods should be applied in testing all examined samples rather than only microscopically positive ones. No *Sarcocystis* spp. were identified in the mucosal scrapings of a single American mink and three European pine martens using species-specific PCR; however, oocysts/sporocysts were detected in these samples under a light microscope. Thus, these animals were most likely infected with oocysts/sporocysts of *Sarcocystis* spp., which employ other than cattle IH. There are a few reports on mustelids as DH for *Sarcocystis* spp. Transmission experiments have shown that mustelids are DH of several *Sarcocystis* spp., *S*. *campestris*, *S*. *muris*, *S*. *putorii*, *S*. *undulati* and *S*. *citellivulpes* (invalid species by Dubey [1]) using members of the order Rodentia as IH [8]. Further studies are needed to reveal the role of mustelids in the transmission of *Sarcocystis* spp. using various mammals and birds as IH. 

*Sarcocystis* spp. identified in the present study, namely, *S. bovifelis, S. cruzi, S. hirsuta* and *S. hominis*, are specific to their IH [32]. Molecular data suggest that *S. cruzi* might occasionally infect water buffaloes (*Bubalus bubalis*) [33]. However, sheep, goats, pigs, horses and other domestic animals raised in Lithuania cannot serve as IH of the above-mentioned *Sarcocystis* spp. [1]. Of the Lithuanian wild fauna, only the European bison (*Bison bonasus*) can possibly act as an IH of some *Sarcocystis* spp. detected in this study [34,35,36]. However, the *B. bonasus* population in Lithuania is not large, it stands at less than 300 individuals and their distribution range does not intersect with the sites of our material on mustelids [9,10,11]. Therefore, it is impossible for *B. bonasus* to be responsible for the high rates of *S. bovifelis* and *S. cruzi* in the intestinal samples of mustelids. 

The forest is considered a primary habitat of two mustelid species, European pine marten and European badger, though they are frequent visitors to the surrounding woodlots, meadows and riversides [9]. The habitat of the American mink is related to water—they inhabit banks of rivers, lakes and ponds. These mustelid species are not closely related to human settlements. Two other investigated mustelids, American mink and European polecat, are more often related to settlements than to other habitats, such as forests and shrubby areas [9]. Habitats preferred by mustelids in Lithuania are similar to those in other countries [37]. Diet peculiarities of the investigated mustelids are not directly related to the involvement of these species in the transmission of *Sarcocystis* spp. using cattle as IH. All the investigated mustelid species are opportunistic feeders. Among such diet sources as fruits, berries and other plant materials, invertebrates, fish, amphibians, birds and various mammals [12,13,14,15,16,17], only one source, namely, cattle carrion, or other sources of cattle meat may be related to *Sarcocystis* spp. we have identified. Mustelid species that we have investigated [12,13,14,15,16,17], with the exception of the American mink [38], use carrion of wild ungulates.

Cattle are too large prey for mustelids to hunt; therefore, mustelids become infected with *S*. *bovifelis*, *S*. *cruzi*, *S*. *hirsuta* and *S*. *hominis* species by scavenging carcasses of cattle. However, habitat distribution of the five investigated mustelid species in Lithuania (see above) should exclude contact with carrion of at least two species, American mink and European pine marten. Therefore, the first assumption about high rates of *Sarcocystis* spp. employing cattle as IH is related to food safety issues. In further studies we are going to examine in what way cattle carcasses or beef waste become accessible to mustelids in Lithuania. It is important to understand whether there are gaps in the management of anthropogenic carrion [39] and if this has already become a source of predictable resources accessible to mustelids. Improper carrion management may be related to (i) dumping sites, (ii) treatment of the waste from meat processing factories, especially small ones and located in the countryside and (iii) raw meat waste from homesteads and farms. The two last sources may be neighboring forests and water bodies, therefore becoming sources of possible infection and available even to the American mink and European pine marten, otherwise having no contact with cattle carrion.

Historically, the disclosure of DH of *Sarcocystis* spp. was performed by transmission experiments [40]. Among carnivorous mammals, transmission experiments of *Sarcocystis* spp. have mainly been carried out with dogs, foxes and cats [41,42]. Recently, molecular methods have been applied for the identification of *Sarcocystis* spp. from fecal or mucosal scraping samples of various wild predators or scavengers infected under natural conditions [2,29,30,31]. The present work is the first study of the molecular identification of *Sarcocystis* spp. in mustelids. Further molecular examination of oocysts/sporocysts detected in the intestine or fecal samples of mustelids can help to clarify the role of these carnivorous mammals in the transmission of *Sarcocystis* parasites. 

It is well known that *Sarcocystis* spp. transmitted via canids cannot be spread via felids and vice versa [1]. However, there is a lack of data on whether *Sarcocystis* spp. transmitted via canids and/or felids can be spread via mustelids. It was demonstrated that mustelids and canids could serve as DH of *S*. *undulati* and *S*. *citellivulpes* [8,43], whereas mustelids and felids could act as DH for *S*. *muris* [8]. Two species, *S*. *bovifelis* (89.3%) and *S*. *cruzi* (73.8%), were most common in the analyzed intestinal samples of mustelids (Figure 2), whereas *S*. *hirusta* and *S*. *hominis* were confirmed in three and single samples, respectively. Canids serve as DH for *S*. *cruzi*, felids act as DH for *S*. *hirsuta* and *S*. *bovifelis* and humans are DH for *S*. *hominis* [19]. Thus, our results indicate that mustelids might be involved in the transmission of *Sarcocystis* spp. which were confirmed to be transmitted via canids and felids. Nevertheless, further detailed studies on this subject are required. Considering a low abundance of wild felids in Lithuania, we speculate that *S*. *hirsuta* is mainly transmitted via felids and *S*. *bovifelis* is mainly transmitted via mustelids. To test the hypothesis, the prevalence of *S*. *hirsuta* and *S*. *bovifelis* in muscles of cattle can be examined in European countries where wild felids are more prevalent. Estonia and Finland are the nearest countries with similar environments and with similar abundances of mustelids but with the high abundances of Eurasian lynx, while Germany or Belgium may be the reference countries with the European wildcat (*Felis silvestris*) populations [44].

## 5. Conclusions

Using a molecular analysis four *Sarcocystis* spp. employing cattle as IH (S. *bovifelis*, *S*. *cruzi*, *S*. *hirsuta* and *S*. *hominis*) were identified in the intestine mucosal scrapings of five Mustelidae species for the first time. Thus, the results of the present study indicate that a wide range of mustelids serve as DH of these *Sarcocystis* spp. Therefore, it is necessary to identify gaps in the management of cattle carrion and beef waste.

## Figures and Tables

**Table 1:** The primers used for the nested PCR.

Species	Primer Name	Primer Sequence	Orientation	The Run of Nested PCR
*S*. *bovifelis*	SF1 ^1^	ATGGCGTACAACAATCATAAAGAA	Forward	First
	SkatR	CAGGCTGAACAGHABTACGA	Reverse	First
	V2bo3	ATATTTACCGGTGCCGTACTTATGTT	Forward	Second
	V2bo4	GCCACATCATTGGTGCTTAGTCT	Reverse	Second
*S*. *cruzi*	SF1 ^1^	ATGGCGTACAACAATCATAAAGAA	Forward	First
	SsunR2	GTGCCTCCCAGGCTGAAYAG	Reverse	First
	GsScruF	TGTATCTACTTACGGCAGGTATCTTT	Forward	Second
	GsScruR	CGTAGTTAGATCCATATCACTCGGTA	Reverse	Second
*S*. *hirsuta*	SF1 ^1^	ATGGCGTACAACAATCATAAAGAA	Forward	First
	SkatR	CAGGCTGAACAGHABTACGA	Reverse	First
	GaHiEF ^2^	GTTGTGCGGTATGAATTATCAACCT	Forward	Second
	GaHiER ^2^	GGTAAGAACTGGAATGGTTAATATCAG	Reverse	Second
*S*. *hominis*	VohoF	GTGCGGTATGAACTGTCTACTGCT	Forward	First
	VohoR	AATACCTGCCCGGCCTTAAC	Reverse	First
	GaHoEF ^2^	TCTCTGGTTTTGGTAACTACTTCGT	Forward	Second
	GaHoER ^2^	CAGACACTGGGATATAATACCGAAC	Reverse	Second

^1^ [24], ^2^ [19].

**Table 2:** Identification of *Sarcocystis* spp. oocysts/sporocysts in mustelids using microscopic and molecular examination.

Host Species	*N*	*Sarcocystis* spp. Positive Animals					
		Microscopic Analysis			Molecular Analysis		
		*n*	%	95% CI	*N*	%	95% CI
American mink	40	15	37.5	24.2–53.0	38	95.0 ***	83.5–98.6
Beech marten	4	3	75.0	30.1–95.4	4	100 ^NS^	51.0–100.0
European pine marten	20	12	60.0	38.7–78.1	15	75.0 ^NS^	53.1–88.8
European badger	5	1	20.0	36.2–62.5	4	80.0 *	37.6–96.4
European polecat	15	9	60.0	35.8–80.2	14	93.3 **	70.2–98.8
Total	84	40	47.6	37.3–58.2	75	89.3 ***	80.9–94.34

Significance of differences between methods is shown in superscript: * *p* < 0.05, ** *p* < 0.01, *** *p* < 0.0001, ^NS^ not significant.

**Table 3:** Intra- and inter-specific genetic variability of identified *Sarcocystis* spp.

*Sarcocystis* spp.	GenBank Accession No. (Length in bp)	Sequence Similarity (%)		
		Comparing Obtained sequences	Comparing Isolates of the Same Species	Comparing Isolates with Other Closely Related Species
*S*. *bovifelis*	MW595468–MW595542 (361)	98.4–100	97.2–100% *S*. *bovifelis* (KT900961–KT900998, KC209690–KC209696, MK962347–MK962348, MT796903–MT796925)	92.5–94.5% *S*. *bovini* (KT900999–KT901022, LC171858)
*S*. *cruzi*	MW595543–MW595604 (556)	98.2–100	96.0–100% *S*. *cruzi* (KC209597–KC209600, KT901078–KT901095, LC171859–LC171862, MG787071–MG787076, MT796926–MT796945)	90.8–93.4% *S*. *pilosa* (KU753903–KU753910, LC349942, LC349966–LC349967, LC466196–LC466201, LC481077–LC481081, LC496070, MT070670– MT070677)
*S*. *hirsuta*	MW595605–MW595607 (461)	98.9–99.8	98.9–99.8% *S*. *hirsuta* (KC209634, KT901023–KT901077, LC171863, MT796946–MT796951, MT796958–MT7969)	95.6–96.3% *S*. *buffalonis* (KU247868–KU247873, MG792800–MG792802)
*S*. *hominis*	MW595608 (501)	-	97.6–99.0% *S*. *hominis* (MH021119, MK497840–MK497843, MT796961–MT796964)	87.1–87.8% *S*. *bovifelis*

## Data Availability

Data supporting the conclusions of this article are included in the article. The sequences generated in the present study were submitted to the GenBank database under accession numbers MW595468–MW595608.
